# Effect of Qigong exercise on non‐motor function and life quality in stroke patients: A systematic review and meta‐analysis

**DOI:** 10.1002/brb3.3246

**Published:** 2023-09-04

**Authors:** Yi Lan, Qiqi You, Qingqing Jiang, Xiaoxiang Peng, Shiyi Cao, Jian Sun

**Affiliations:** ^1^ Wushu College Wuhan Sports University Wuhan China; ^2^ School of Public Health, Tongji Medical College Huazhong University of Science and Technology Wuhan Hubei China; ^3^ Department of Neurology, the Third People's Hospital of Hubei Province Jianghan University Wuhan China; ^4^ Northeast China Ethnic Traditional Sports Research Center Wuhan Sports University Wuhan China

**Keywords:** meta‐analysis, non‐motor function, Qigong, stroke

## Abstract

**Background:**

Qigong have a positive impact on the rehabilitation of motor function in stroke survivors, but there is no consensus on the effectiveness of Qigong on activities of daily living (ADL), neurological function, and life quality for patients with stroke. We aimed to quantitatively evaluate the effect of Qigong on non‐motor function and life quality in stroke patients.

**Methods:**

Electronic literature searches were performed for randomized controlled trials on this topic using PubMed and China National Knowledge Infrastructure through August 2022. The primary outcome measures were the Barthel Index, neurological deficit score (NDS), and Stroke‐Specific Quality of Life Scale (SSQLS). A random‐effects model was used to calculate the pooled mean difference (MD) with 95% confidence interval (CI). RevMan 5.4 software was used for meta‐analysis.

**Results:**

A total of 16 eligible randomized controlled trials with 1253 stroke patients were included. As indicated by the Barthel Index, Qigong was associated with the improvement in daily living activities of stroke patients (MD: 10.72, 95% CI: 5.88∼15.57). It was also found that Qigong was helpful in improving life quality (SSQLS, MD: 14.41, 95% CI: 5.56∼23.25) and reducing NDSs among them (NDS, MD: −4.56, 95% CI: −6.99∼−2.14). After sensitivity analysis, the effect of Qigong on these functions and life quality did not change significantly. By subgroup analysis of intervention duration, we found that long‐term intervention (MD: 11.83, 95% CI: 2.80∼20.86) had a better effect on the improvement of daily living activities than short‐term intervention (MD: 10.07, 95% CI: 6.15∼14.00) (*p_for subgroup differences_* = .001).

**Conclusions:**

Pooled results suggested that Qigong had beneficial effects on ADL, neurological function, and life quality in stroke patients, which may provide an option for their rehabilitation.

## Graphical abstract

Stroke‐induced dysfunction is associated with reduced life quality for stroke survivors and places a heavy burden on stroke families

• Qigong has been suggested to be a potentially viable form of complementary and alternative medicine, but comprehensive evidence to support this is lacking in stroke patients

• This meta‐analysis supports the beneficial effects of Qigong on daily living activities, neurological function, and life quality in stroke patients, which may provide an option for the rehabilitation of stroke patients.

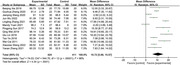

## 1 INTRODUCTION

Stroke is a leading cause of disability worldwide, with 143 million years of disability‐adjusted life reported in 2019 due to it (Collaborators GBDS, 2021). Half of stroke survivors are disabled, and a third of them rely on others for help in their activities of daily living (ADL; Markus, 2022), which is associated with reduced quality of life for stroke survivors and places a significant burden on stroke families, especially those with low economic levels (Buvarp et al., 2020). It is estimated that this burden will further increase as a growing and aging population is likely to bring more disabled stroke survivors (Stinear et al., 2020). Therefore, it is necessary to find an economical, effective, and safe rehabilitation alternative to improve the function and life quality of stroke survivors.

Qigong mainly includes Wuqinxi, Liuzijue, Baduanjin, and Yijinjing, which are characterized by slow and gentle movements and regulation of consciousness and breathing. It is now also gaining popularity abroad because of the wide distribution of Chinese around the world and its mysterious effects to enhance joint flexibility, balance, and neurological flexibility (Klein et al., 2016; Koh, 1982) There is increasing clinical evidence that Qigong was helpful for the improvement of lung function (Xu et al., 2022), the life quality, and other symptoms in patients with chronic disease (Gouw et al., 2019). Several reviews have also evaluated the effect of Qigong on the rehabilitation of stroke patients. However, they mainly focused on the motor function of stroke patients (Chen et al., 2015; Ge et al., 2017; Zou et al., 2018), and comprehensive evidence evaluating the effect of Qigong on other functions of stroke patients was lacking.

Evidence suggested that the benefits of Qigong on human health made it a potentially viable form of complementary and alternative medicine (Jahnke et al., 2010), but whether this is the case in stroke patients needs further testing. Therefore, we conducted a meta‐analysis to evaluate the possible effects of Qigong exercise on ADL, neurological deficit score (NDS), and quality of life in stroke survivors.

## 2 METHODS

This study has been registered with PROSPERO on September 2, 2022, and the registration number was CRD42022355179 (https://www.crd.york.ac.uk/prospero/).

### 2.1 Literature search

Electronic literature searches were performed using PubMed and China National Knowledge Infrastructure for randomized controlled trials evaluating the effect of Qigong exercise in stroke patients through August 2022. The search strategy combined terms about the intervention (Qigong or Wuqinxi or Liuzijue or Baduanjin or Yijinjing) and terms about the disease (stroke). Qigong, Wuqinxi, Liuzijue, Baduanjin, and Yijinjing are all different forms of traditional Chinese health exercises that focus on cultivating both physical and mental well‐being. Qigong is a broad term that encompasses various practices, while Wuqinxi, Liuzijue, Baduanjin, and Yijinjing are specific qigong exercises with unique characteristics. Wuqinxi emphasizes imitating animal movements, Liuzijue focuses on specific sounds and breath control, Baduanjin consists of eight exercises, and Yijinjing emphasizes muscle and tendon conditioning (Supporting Information S1). Articles in English or Chinese were both considered. Reference lists of some review articles were manually searched to avoid missing relevant articles.

### 2.2 Eligibility criteria

Randomized controlled trials that met the following eligibility criteria would be included. (1) They were conducted among patients with a definite diagnosis of stroke; (2) the primary intervention in the intervention group was Qigong exercise (including Wuqinxi, Liuzijue, Baduanjin and Yijinjing); (3) the control group received no intervention, routine intervention, or other active intervention; (4) outcomes considered in this systematic review included ADL, neurological score, and life quality; and (5) data were provided to calculate the mean difference (MD) with 95% confidence intervals (95% CIs) for each outcome measure.

The stroke survivors’ ADL was assessed using the Barthel Index scale, which consisted of 10 items (score range: 0–100), with a higher score indicating greater independence in performing ADL (McGill et al., 2022). The stroke survivors’ neurological function was assessed using the NDSs, which consisted of eight items (score range: 0–45), with a lower score indicating greater neurological function (Shao et al., 2021). The stroke survivors’ life quality was assessed using the Stroke‐Specific Quality of Life Scale (SSQLS), which consisted of 49 items (score range: 49–245), with higher scores indicating better quality of life (Muus et al., 2007).

### 2.3 Study selection and data extraction

Study eligibility was assessed independently by two reviewers. They then extracted data on first author, publication year, country, language, participants, sample size, methods, frequency, and duration of intervention for experimental and control groups, mean values and standard deviations of post‐intervention outcome of the experimental group and control group, and measures of outcome, with disagreements solved by consultation to reach consensus.

### 2.4 Risk of bias assessment

We assessed the methodological quality of included randomized controlled trials using the Cochrane collaboration's tool for assessing risk of bias (Cochrane Handbook for Systematic Reviews of Interventions, Version 6.4, 2023). Each study was evaluated in seven aspects, including random sequence generation, allocation concealment, blinding of participants and personnel, blinding of outcome assessment, incomplete outcome data, selective reporting, and other bias, and each aspect was recoded as “low risk,” “unclear risk,” or “high risk.”

### 2.5 Statistical analysis

The effect size and corresponding 95% CI of the post‐intervention score for each outcome and study were calculated. The MD was calculated and pooled by a fixed or a random effect model according to the chi‐square and *I* ^2^ tests, which were used to measure statistical heterogeneity. When the *p*‐value was less than .1 and *I* ^2^ was less than or equal to 50%, a fixed effect model was used; otherwise, a random effect model was applied.

In some of the included randomized controlled trials, the participants completed the outcome assessments two to three times throughout the study periods. Thus, we only extracted the data of assessment results, which compared the experimental group and control group for the full study duration (Lin, 2018; Wei & Zhang, 2019; Yuen et al., 2021; G. Zhang et al., 2017). Sensitivity analysis was performed with the one‐study‐exclusion strategy to test the stability of the combined results. We divided these studies into different subgroups to explore the possible influence of intervention duration (short‐term: ≤12 weeks vs. long‐term: >13 weeks) and types of Qigong on the effect size. Begg's test and Egger's test were conducted to detect the existence of publication bias, and the trim‐and‐fill method was used to correct publication bias (Duval & Tweedie, 2000). All statistical analyses were performed using the Review Manager 5.4 software.

## 3 RESULTS

### 3.1 Literature search

**FIGURE 1:**
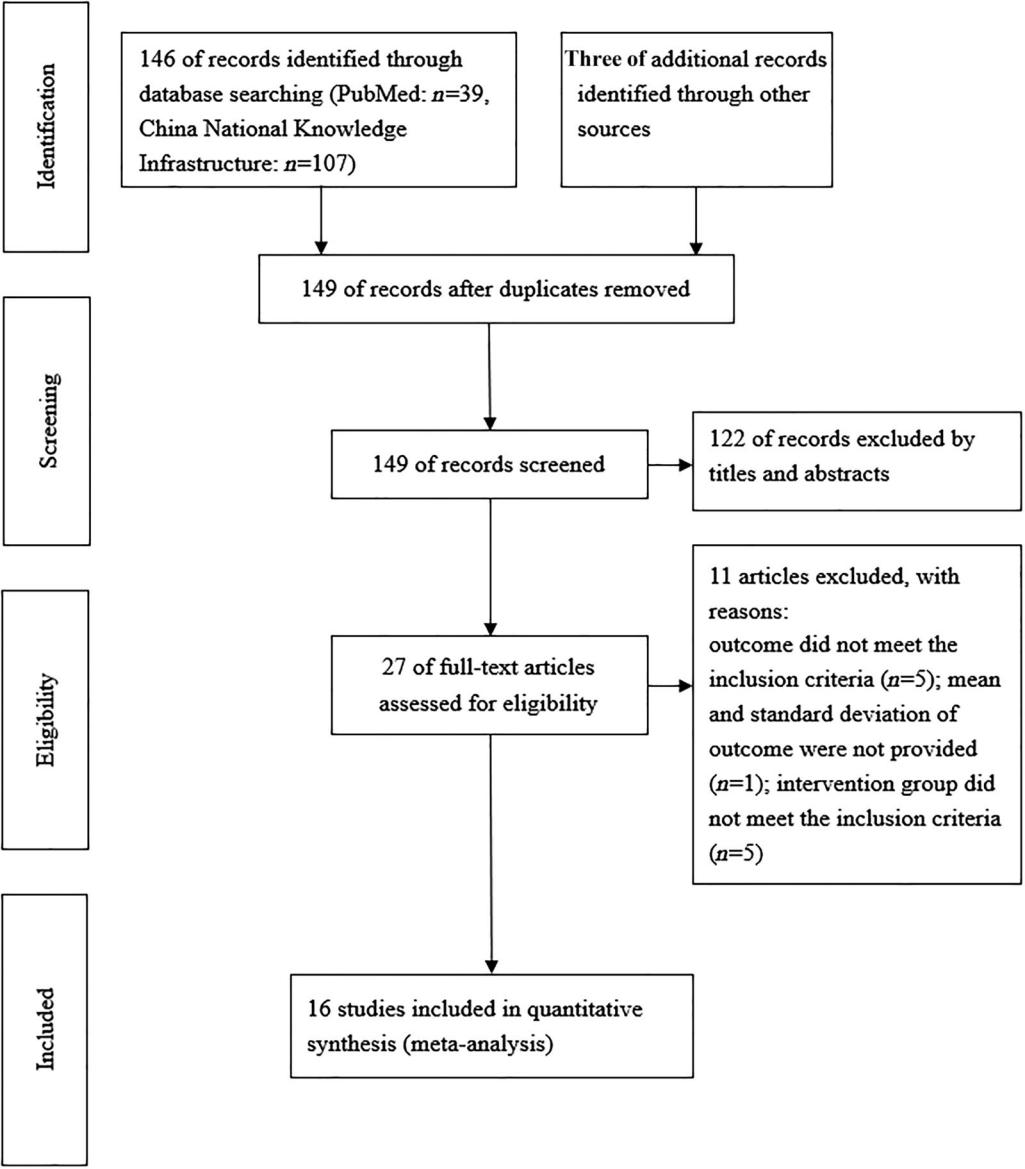
Study screening and identification flow diagram.

The complete process of literature search and identification is shown in Figure 1. A total of 146 records were retrieved from the electronic database using a prespecified search strategy, and three additional records were retrieved through other sources. By reviewing the title and abstract, 122 records that did not meet the criteria were excluded, leaving 27 full‐text articles to be evaluated for eligibility. Of them, 11 articles were further excluded for the following reasons: outcome that did not meet the inclusion criteria (*n* = 5); mean and standard deviation of outcome that were not provided (*n* = 1); and the intervention group that did not meet the inclusion criteria or completely unrelated to Qigong exercise (*n* = 5). Finally, 16 randomized controlled trials were included for the meta‐analysis.

### 3.2 Characteristics and quality of included randomized controlled trials

Supporting Information S2 presented the basic characteristics of the included randomized controlled trials. The number of participants ranged from 40 to 224, with an average age of over 50 years old, and the total number of participants across all randomized controlled trials was 1253. All the randomized controlled trials were conducted in China, and three of them were reported in English (Yuen et al., 2021; G. Zheng et al., 2020; Y. Zheng et al., 2021). Of the 16 randomized controlled trials, 12 adopted Baduanjin as the primary intervention (Cai & Liang, 2011; Guo et al., 2013; Lin, 2018; J. Wang et al., 2020; X. Wang et al., 2022; Wei & Zhang, 2019; Xie et al., 2019; Ye et al., 2018; Yuen et al., 2021; L. Zhang & Huang, 2021; G. Zheng et al., 2020; Zhou et al., 2021), two adopted Yijinjing as the primary intervention (Sun et al., 2017; G. Zhang et al., 2017), and two adopted Liuzijue as the primary intervention (J. Wu et al., 2022; Y. Zheng et al., 2021). The shortest intervention duration was 3 weeks and the longest was 6 months. Thirteen randomized controlled trials assessed the effect on daily living activities, four on neurological function, and three on life quality.

**FIGURE 2:**
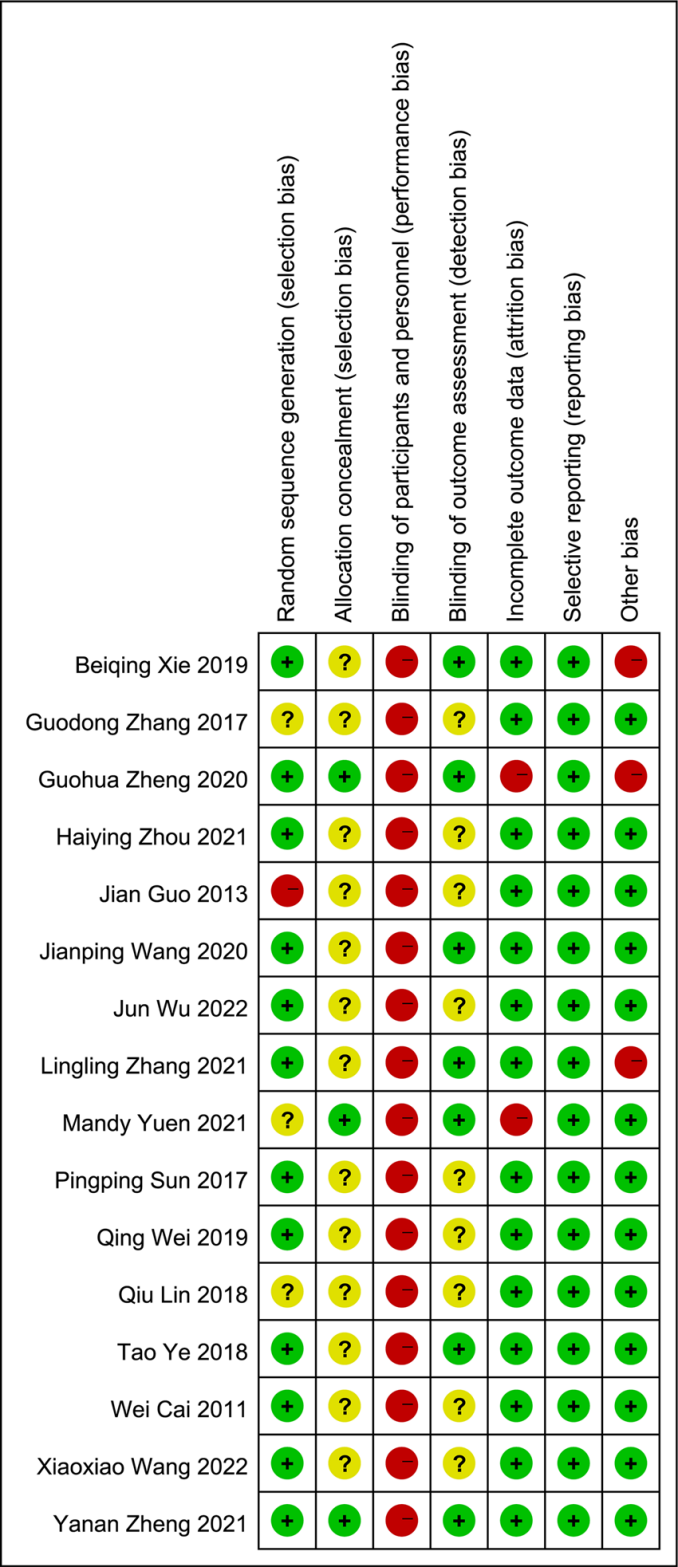
Risk of bias summary.

The risk of bias assessment result are shown in Figure 2. Of the 16 randomized controlled trials, 12 (75.0%) reported the methods of random sequence generation, mainly through the random number table method, and only three (18.8%) described allocation concealment methods. Because it was difficult to blind participants and personnel in exercise therapy, all studies were of high risk regarding to “blinding of participants and personnel.” Six studies (37.5%) blinded the outcome assessors, and the remaining were unclear. Fourteen studies had no or low numbers of dropouts, while two studies reported dropout rates of about 14.0%. All but two of the studies had sample sizes above 50, and all studies reported homogeneity of the baseline data.

### 3.3 Effects of Qigong exercise on non‐motor function and life quality in stroke patients

#### 3.3.1 ADL

**FIGURE 3:**
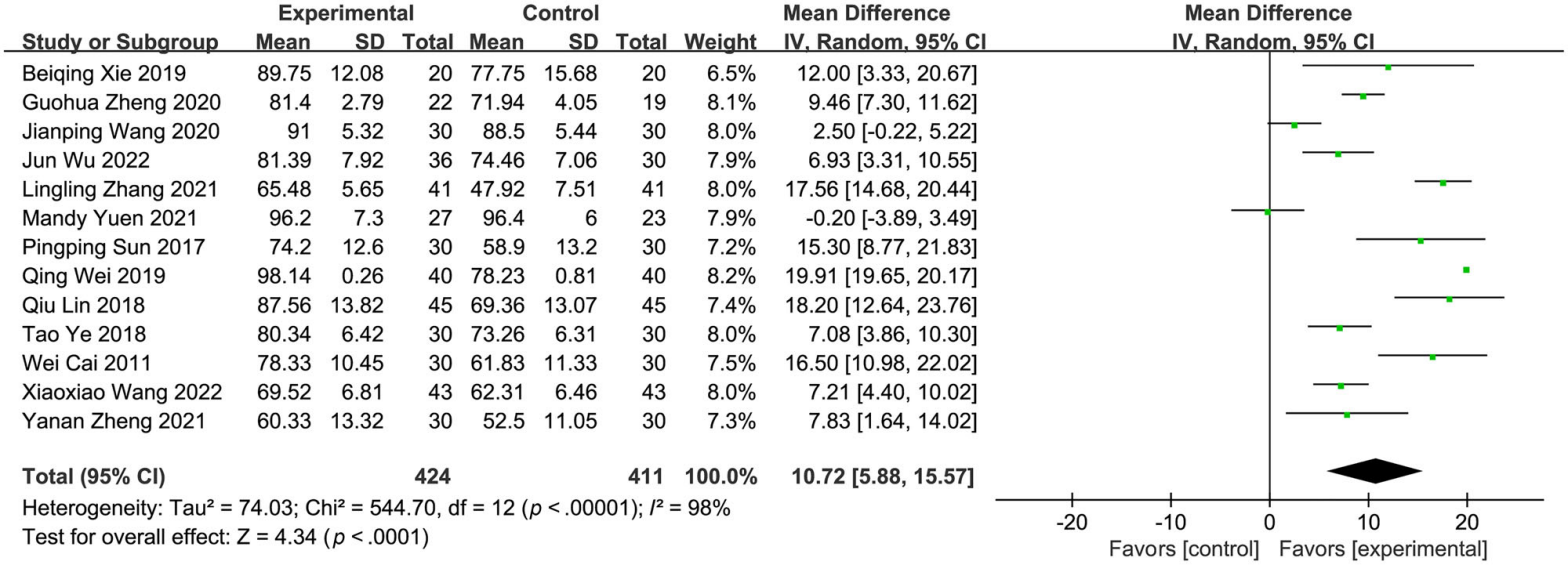
Forest plot of the effect of Qigong on daily living activities in stroke patients.

Of the 13 randomized controlled trials with ADL as the outcome (Cai & Liang, 2011; Lin, 2018; Sun et al., 2017; J. Wang et al., 2020; X. Wang et al., 2022; Wei & Zhang, 2019; J. Wu et al., 2022; Xie et al., 2019; Ye et al., 2018; Yuen et al., 2021; L. Zhang & Huang, 2021; G. Zheng et al., 2020; Y. Zheng et al., 2021), the Barthel Index scale were used as the outcome measure (higher scores indicated better ability—Hsieh et al., 2007; and the minimal clinically important difference of the Barthel Index scale was classified as an improvement within the experiential group by 9.25 points). Pooled results indicated that Qigong exercise could improve the daily living activities of stroke patients (MD: 10.72, 95% CI: 5.88∼15.57, *I* ^2^= 98%; Figure 3). After deleting the study of Wei et al. (Wei & Zhang, 2019) in sensitivity analysis, the MD changed to 9.73 (95% CI: 6.41∼13.06), with the heterogeneity reduced to 90%.

#### 3.3.2 Neurological score

**FIGURE 4:**
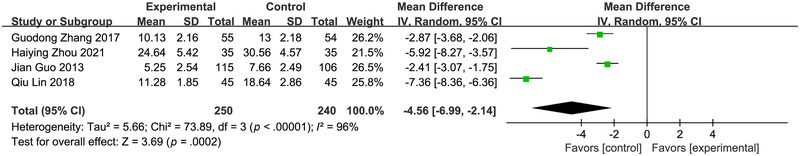
Forest plot of the effect of Qigong on neurological score in stroke patients.

Four randomized controlled trials (Guo et al., 2013; Lin, 2018; L. Zhang et al., 2017; Zhou et al., 2021) used the NDS to assess neurological function in stroke patients (lower scores indicated better neurological function (Shao et al., 2021). Pooled results showed that Qigong exercise was helpful in reducing NDSs of stroke patients (MD: −4.56, 95% CI: −6.99∼−2.14, *I* ^2^= 96%; Figure 4). After the study of Lin et al. (Lin, 2018) was excluded, the heterogeneity decreased to 75% (MD: −3.19, 95% CI: −4.40∼−1.99).

#### 3.3.3 Quality of life

**FIGURE 5:**
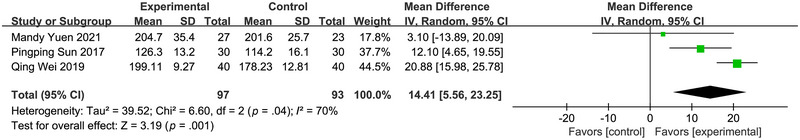
Forest plot of the effect of Qigong on life quality in stroke patients.

Three randomized controlled trials (Sun et al., 2017; Wei & Zhang, 2019; Yuen et al., 2021) evaluated the effect of Qigong exercise on quality of life in stroke patients by SSQLS (higher scores indicated better quality of life (Muus et al., 2007). It was found that Qigong intervention was associated with a significant improvement in the quality of life in stroke patients (MD: 14.41, 95% CI: 5.56∼23.25, *I* ^2^= 70%; Figure 5). The heterogeneity of combined results was significantly reduced when the study of Wei et al. (Wei & Zhang, 2019) was excluded (MD: 10.65, 95% CI: 3.83∼17.47, *I* ^2^= 0%).

### 3.4 Subgroup analysis and publication bias

Considering the low statistical power when the number of studies included was small, we performed subgroup analysis and publication bias test only on the outcome “ADL.” By subgroup analysis of intervention duration, we found that short‐term and long‐term intervention had different effects (*p_for subgroup differences_* = .001), with long‐term intervention having a better effect on the improvement of daily living activities (short‐term, MD: 10.07, 95% CI: 6.15∼14.00; long‐term, MD: 11.83, 95% CI: 2.80∼20.86; Table 1). Begg's test did not find publication bias (*p* = .067), but Egger's test showed it (*p* < .001). To avoid the possible effect of publication bias, we used the trim‐and‐fill method for correction, and the corrected MD was 1.20 (95% CI: 0.32∼2.08; Supporting Information S3).

**TABLE 1:** Subgroup analysis of the effect of Qigong exercise on daily living activities.

Group	Number of trials	Number of participants	Mean difference (95% confidence interval)	*I* ^2^	Test for subgroup differences
Intervention duration					*p* = .001
Long‐term	4	261	11.83 (2.80, 20.86)	99%	
Short‐term	9	574	10.07 (6.15, 14.00)	89%	
Types of Qigong					*p* = .03
Baduanjin	10	649	10.96 (5.40, 16.52)	98%	
Liuzijue	2	126	7.16 (4.04, 10.28)	0%	
Yijinjing	1	60	15.30 (8.77, 21.83)	–	

### 3.5 Reports of adverse event

Three of these randomized controlled trials had reported dropped cases (Guo et al., 2013; Yuen et al., 2021; G. Zheng et al., 2020), while none of them reported adverse events during the intervention.

## 4 DISCUSSION

Findings of this review indicated that Qigong exercise had beneficial effects on ADL, neurological function, and quality of life in stroke patients. Sensitivity analysis confirmed the stability of the results. Randomized controlled trials included did not report adverse events, which reflected the safety of Qigong exercise for stroke patients to some extent. Considering the above advantages, Qigong may play a further role in the rehabilitation of stroke patients in the future.

Our results were similar to those of previous reviews, which validated the beneficial effect of Qigong on the rehabilitation of stroke patients. In the review of Ge et al. (2017), pooled results showed that traditional Chinese exercises produced positive effects on ADL ability and neurological impairment. However, they included studies on a variety of exercises including Qigong, Tai chi, and Daoyin, which were inevitably heterogeneous, even though they all belonged to traditional Chinese exercises. Zou et al. (2018) evaluated the effect of Baduanjin on stroke rehabilitation, but due to the limited number of studies included, they conducted a qualitative evaluation on neurological deficits, ADL, and quality of life. Given the possible limitations of previous reviews, we conducted a quantitative evaluation of the available evidence and focused only on Qigong exercise.

As a traditional Chinese fitness exercise, Qigong is used by a large number of individuals. For example, middle‐aged and elderly people usually practice Qigong to prevent chronic diseases (Gouw et al., 2019); and in Fangcang Hospital in Wuhan, patients with mild Coronavirus Disease 2019 (COVID‐19) practice Baduanjin for treatment and exercise under the guidance of traditional Chinese medicine doctors (Feng et al., 2020). The mechanism of Qigong acting on human bodies seems mysterious, but there are certain theoretical bases for its effect on health. The improvement effect of Qigong on non‐motor function of stroke patients may be attributed to two aspects. On the one hand, Qigong is helpful for emotion and stress management, on the other hand, regular Qigong exercise could strengthen certain muscles of stroke patients and improve their self‐care ability.

It is well known that the emotions of stroke survivors have a significant impact on disease recovery. It has been suggested that Qigong could enhance non‐reactivity to aversive thoughts and impulses by focusing attention on interoceptive sensations associated with breathing or other parts of the body (Yeung et al., 2018). The study of Hoon Ryu et al. found that the level of some stress hormones in men decreased after Qigong training (Ryu et al., 1996). Additionally, specific muscle groups of the trainer would be strengthened with different movements through Qigong training. Findings of C. Wang et al. (2022) suggested that there was a moderate effect of Qigong on handgrip strength among older adults. In patients with chronic obstructive pulmonary disease, Liuzijue exercise also showed beneficial effects on respiratory muscle strength and peripheral skeletal muscle function (W. Wu et al., 2018).

Sensitivity analyses showed the stability of combined results. With regard to the outcome “quality of life,” heterogeneity of summarized results was significantly reduced after excluding the study by Wei et al. (Wei & Zhang, 2019). This might be because the intervention duration in this study (6 months) was significantly longer than that in the other two studies (Sun et al., 2017; Yuen et al., 2021; 16 weeks and 3 weeks, respectively). Thus, the effect was more pronounced, which partly explained the source of heterogeneity. Subgroup analysis on intervention duration of daily living activities also showed that long‐term interventions were more effective than short‐term interventions. As a result, stroke patients may benefit more from long‐term Qigong training.

### 4.1 Strengths and limitations

Compared with previous reviews on the same topic that carried out qualitative summaries, this review adopted a quantitative synthesis method to present the results. In addition, we focused on the independent effect of Qigong exercise rather than the overall effect of traditional Chinese exercise on stroke patients, which reduced the heterogeneity caused by intervention types to a certain extent.

However, some limitations should be acknowledged. First, it was difficult to blind participants and personnel due to the specificity of the intervention. But this was a common concern in exercise therapy research and was generally unlikely to influence the outcomes (Ge et al., 2017). Second, although we searched English literature, only three articles were retrieved, and they were conducted in China. Therefore, external validity may be somewhat limited, as the participants were predominantly Chinese. Considering the internationalization trend of Chinese traditional sports, the effect of Qigong exercise on the rehabilitation of stroke patients may be further verified in other countries in the future.

## 5 CONCLUSION

Existing evidence suggested that Qigong exercise had beneficial effects on ADL, neurological function, and quality of life in stroke patients. The effect of long‐term Qigong training on ADL was better than that of short‐term training. Our results may provide an option for the rehabilitation of non‐motor functions in stroke patients. More randomized controlled trials are needed to verify the clinical effectiveness of Qigong training in populations from other countries.

## AUTHOR CONTRIBUTIONS

**Yi Lan**: Methodology; formal analysis; writing—original draft. **Qiqi You**: Methodology; formal analysis; writing—review and editing. **Qingqing Jiang**: Conceptualization; writing—review and editing; **Xiaoxiang Peng**: Writing—review and editing. **Shiyi Cao**: Writing—review and editing. **Jian Sun**: Conceptualization; methodology; supervision. All authors critically revised the manuscript and gave final approval of the article.

## CONFLICT OF INTEREST STATEMENT

The authors declare that they have no competing interests.

### PEER REVIEW

The peer review history for this article is available at https://publons.com/publon/10.1002/brb3.3246.

## Supporting information

Supporting Information S1 Distinctions and similarities between these interventions.

Supporting Information S2 Basic characteristics of included studies.

Supporting Information S3 Filled funnel plot of the effect of Qigong on daily living activities.

## Data Availability

The datasets supporting the conclusions of this article are included within the article and its additional files.
