# Supplementary material for: Effect of Qigong exercise on non‐motor function and life quality in stroke patients: A systematic review and meta‐analysis
Source: Brain Behav. 2023 Sep 4;13(11):e3246. doi: 10.1002/brb3.3246 (PMC10636391; doi:10.1002/brb3.3246)
Supplement: Supplementary file 1 — Supporting Information S1 Distinctions and similarities between these interventions. [file BRB3-13-e3246-s002.docx]

Qigong, Wuqinxi, Liuzijue, Baduanjin, and Yijinjing are all different forms of traditional Chinese health exercises that focus on cultivating both physical and mental well-being. Although they share similarities, each intervention has its distinct characteristics and practices. Here’s a description of their distinctions and similarities:

**1. Qigong:**

Qigong is a holistic system that combines movement, meditation, and regulated breathing exercises. It aims to cultivate and balance the body’s vital energy or “qi.” It encompasses a wide range of practices, including gentle movements, postures, and mental focus. Qigong exercises can be categorized into dynamic qigong (moving exercises) and static qigong (meditation and breathing exercises).

**2. Wuqinxi:**

Wuqinxi, also known as “Five Animal Frolics,” is a qigong exercise that imitates the movements and characteristics of five animals: tiger, deer, bear, monkey, and bird. Each animal’s movements correspond to specific physical and energy benefits. Wuqinxi combines stretching, breathing, and mental concentration to promote flexibility, strength, balance, and vitality.

**3. Liuzijue:**

Liuzijue, which means “Six Healing Sounds,” focuses on regulating and harmonizing the body’s internal organs through specific breathing techniques and vocalizations. It involves combining six different sounds with movements that correspond to specific organs, such as liver, heart, spleen, lungs, kidneys, and triple burner. Liuzijue aims to enhance organ functions, improve circulation, and release stress.

**4. Baduanjin:**

Baduanjin, also known as the “Eight Brocades” or “Eight Pieces of Silk,” is a series of eight qigong exercises that date back over a thousand years. Each exercise targets specific areas of the body and aims to improve physical strength, flexibility, and overall health. The movements are gentle and rhythmic, synchronized with regulated breathing and mental focus.

**5. Yijinjing:**

Yijinjing, translated as “Muscle and Tendon Changing Classic,” focuses on both physical and mental conditioning. It consists of a series of repetitive stretching, twisting, and bending movements that aim to strengthen and condition muscles, tendons, and joints. Yijinjing exercises also involve deep breathing, concentration, and visualization techniques to enhance mental focus and clarity.

**Distinctions:**

• Qigong is a broad term that encompasses various practices, while Wuqinxi, Liuzijue, Baduanjin, and Yijinjing are specific qigong exercises with unique characteristics.

• Wuqinxi emphasizes imitating animal movements, Liuzijue focuses on specific sounds and breath control, Baduanjin consists of eight exercises, and Yijinjing emphasizes muscle and tendon conditioning.

• The breathing techniques, movements, and mental aspects differ among these interventions, leading to distinct effects on the body and mind.

**Similarities:**

• All interventions promote the cultivation and balance of qi or vital energy.

• They combine physical movements, regulated breathing, and mental focus.

• These exercises aim to improve health, enhance energy flow, reduce stress, and promote overall well-being.

• They have deep roots in Chinese traditional medicine and philosophy.

It’s worth noting that while these interventions share similarities and have proven benefits for many individuals, it’s essential to consult with a qualified instructor or healthcare professional to ensure proper practice and to adapt to individual needs.
