# Supplementary material for: Effect of Qigong exercise on non‐motor function and life quality in stroke patients: A systematic review and meta‐analysis
Source: Brain Behav. 2023 Sep 4;13(11):e3246. doi: 10.1002/brb3.3246 (PMC10636391; doi:10.1002/brb3.3246)
Supplement: Supplementary file 2 — Supporting Information S2 Basic characteristics of included studies. [file BRB3-13-e3246-s003.docx]

**Additional file 1** Basic characteristics of included studies

| Studyid | Participants | Sample size (age, years) | Interventions | Duration & Frequency of trials period | Measures of outcome |
| --- | --- | --- | --- | --- | --- |
| Qiu Lin 2018 | Patients of stroke at the recovery stage | 90 (Ex: 68.82 ± 3.91; Co: 68.32 ± 3.73) | Ex: Baduanjin + traditional Chinese medicine; Co: traditional Chinese medicine | 6 months, 30 min each time, 1 time per  day | Barthel Index, Neurological deficit score |
| Qing Wei 2019 | Convalescent stroke patients | 80 (Ex: 56.1 ± 9.2; Co: 58.7 ± 10.3) | Ex: Baduanjin + conventional Western medicine; Co: conventional Western medicine | 6 months, 30 min each time, 5 times per week | Barthel Index, Stroke-Specific Quality of Life scale |
| Tao Ye 2018 | Patients of stroke at the recovery stage | 60 (Ex: 59.81 ± 7.54; Co: 60.17 ± 7.21) | Ex: Baduanjin + routine rehabilitation and cluster needling technique; Co: routine rehabilitation and cluster needling technique | 6 weeks, 20 min each time, 2 times per  day, 5 days per week | Barthel Index |
| Guohua Zheng 2020 | Stroke survivors with cognitive impairment | 48 (Ex: 61.63 ± 9.21; Co: 62.75 ± 6.41) | Ex: Baduanjin + original medication and rehabilitation; Co: original medication and rehabilitation | 24 weeks, 40 minutes per day, 3 days per week | Barthel Index |
| Mandy Yuen 2021 | Convalescent stroke patients | 58 (Ex: 63.1 ± 10.6; Co: 62.0 ± 13.1) | Ex: Baduanjin; Co: conventional fitness training | 16 weeks, 50 minutes per session, 3 days per  week | Barthel Index, Stroke-Specific Quality of Life scale |
| Yanan Zheng 2021 | Convalescent stroke patients | 60 (Ex: 63.50 ± 10.36; Co: 67.23 ± 9.15) | Ex: Liuzijue + conventional rehabilitation; Co: conventional respiration training + conventional rehabilitation | 3 weeks, 45 minutes per session, 5 times per week | Barthel Index |
| Pingping Sun 2017 | Convalescent stroke patients | 60 (Ex: 63.73 ± 6.37; Co: 64.37 ± 5.74) | Ex: Yijinjing + routine rehabilitation; Co: routine rehabilitation | 3 weeks, 40 minutes per time, 3 times per week | Barthel Index, Stroke-Specific Quality of Life scale |
| Beiqing Xie 2019 | Convalescent stroke patients | 40 (Ex: 51.10 ± 12.92; Co: 53.95 ± 13.00) | Ex: Baduanjin + conventional rehabilitation; Co: conventional rehabilitation | 3 weeks, 25 minutes per time, 5 days per week | Barthel Index |
| Lingling Zhang 2021 | Elderly stroke patients with hemiplegia | 82 (Ex: 71.29 ± 4.51; Co: 70.45 ± 4.29) | Ex: Baduanjin + routine rehabilitation; Co: routine rehabilitation | 8 weeks, 3 times per day, 5 days per week | Barthel Index |
| Wei Cai 2011 | Patients with sequelae of stroke | 60 (Ex: 60.27 ± 10.48; Co: 61.27 ± 7.42) | Ex: Baduanjin + routine health guidance; Co: routine health guidance | 3 months, 30 minutes per time, 4-5 times per week | Barthel Index |
| Jun Wu 2022 | Post-stroke patients with cardiopulmonary dysfunction | 66 (Ex: 63.05 ± 5.79; Co: 63.52 ± 5.81) | Ex: Liuzijue + routine cardiopulmonary training; Co: routine cardiopulmonary training | 12 weeks, 30 minutes per day, 5 times per week | Barthel Index |
| Jianping Wang 2020 | Patients with post-stroke fatigue | 60 (Ex: 55.10 ± 6.28; Co: 55.97 ± 6.21) | Ex: Baduanjin + routine rehabilitation; Co: free movement + routine rehabilitation | 4 weeks, 12 minutes per day, 5 days per week | Barthel Index |
| Guodong Zhang 2017 | Convalescent stroke patients | 109 (Ex: 69.21 ± 7.30; Co: 65.81 ± 8.20) | Ex: Yijinjing + acupuncture treatment; Co: acupuncture treatment | 12 weeks, 30 minutes per day, 6 days per week | Neurological deficit score |
| Jian Guo 2013 | Convalescent stroke patients | 221 (33-82) | Ex: Baduanjin + basic drug treatment and rehabilitation; Co: basic drug treatment and rehabilitation | 6 weeks, 40 minutes per day, 7 days per week | Neurological deficit score |
| Haiying Zhou 2021 | Elderly patients with convalescent cerebral vascular accident | 70 (Ex: 69.1 ± 8.5; Co: 69.5 ± 8.3) | Ex: Baduanjin + routine rehabilitation; Co: routine rehabilitation | 3 months, 60 minutes per time, 5 times per week | Neurological deficit score |
| Xiaoxiao Wang 2022 | Stroke patients with hemiplegia | 86 (Ex: 54.52 ± 7.36; Co: 56.28 ± 8.54) | Ex: Baduanjin + body function exercise; Co: body function exercise | 6 weeks, 50 minutes per day, 5 days per week | Barthel Index |

RCT: randomized controlled trial; Ex: experimental group; Co: control group
