# Supplementary figures and images for: Effect of Qigong exercise on non‐motor function and life quality in stroke patients: A systematic review and meta‐analysis
Source: Brain Behav. 2023 Sep 4;13(11):e3246. doi: 10.1002/brb3.3246 (PMC10636391; doi:10.1002/brb3.3246)

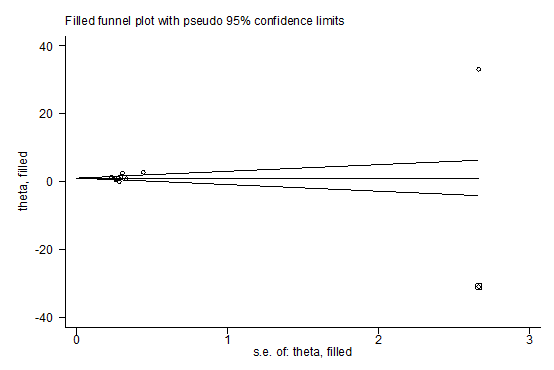

Supplement: Supplementary file 3 — Supporting Information S3 Filled funnel plot of the effect of Qigong on daily living activities. [file BRB3-13-e3246-s001.tif]
